# Active vaccination with vaccinia virus A33 protects mice against lethal vaccinia and ectromelia viruses but not against cowpoxvirus; elucidation of the specific adaptive immune response

**DOI:** 10.1186/1743-422X-10-229

**Published:** 2013-07-10

**Authors:** Nir Paran, Shlomo Lustig, Anat Zvi, Noam Erez, Tomer Israely, Sharon Melamed, Boaz Politi, David Ben-Nathan, Paula Schneider, Batel Lachmi, Ofir Israeli, Dana Stein, Reuven Levin, Udy Olshevsky

**Affiliations:** 1 Department of Infectious Diseases, Israel Institute for Biological Research, P.O. box 19, Ness-Ziona 74100, Israel; 2 Department of Biochemistry and Molecular Biology, Israel Institute for Biological Research, P.O. box 19, Ness-Ziona 74100, Israel

**Keywords:** Smallpox, Subunit vaccine, A33, Cowpox, Vaccinia, Monkeypox, 1G10, Alphavirus, Sindbis, In-vivo protection

## Abstract

Vaccinia virus protein A33 (A33^VACV^) plays an important role in protection against orthopoxviruses, and hence is included in experimental multi-subunit smallpox vaccines. In this study we show that single-dose vaccination with recombinant Sindbis virus expressing A33^VACV^, is sufficient to protect mice against lethal challenge with vaccinia virus WR (VACV-WR) and ectromelia virus (ECTV) but not against cowpox virus (CPXV), a closely related orthopoxvirus. Moreover, a subunit vaccine based on the cowpox virus A33 ortholog (A33^CPXV^) failed to protect against cowpox and only partially protected mice against VACV-WR challenge. We mapped regions of sequence variation between A33^VACV^ and A33^CPXV^and analyzed the role of such variations in protection. We identified a single protective region located between residues 104–120 that harbors a putative H-2Kd T cell epitope as well as a B cell epitope - a target for the neutralizing antibody MAb-1G10 that blocks spreading of extracellular virions. Both epitopes in A33^CPXV^ are mutated and predicted to be non-functional. Whereas vaccination with A33^VACV^ did not induce *in-vivo* CTL activity to the predicted epitope, inhibition of virus spread *in-vitro*, and protection from lethal VACV challenge pointed to the B cell epitope highlighting the critical role of residue L118 and of adjacent compensatory residues in protection. This epitope’s critical role in protection, as well as its modifications within the orthopoxvirus genus should be taken in context with the failure of A33 to protect against CPXV as demonstrated here. These findings should be considered when developing new subunit vaccines and monoclonal antibody based therapeutics against orthopoxviruses, especially variola virus, the etiologic agent of smallpox.

## Introduction

Variola virus, the causative agent of smallpox, was eradicated following a world-wide vaccination campaign, launched by the WHO [1]. Highly conserved antigens (>97% sequence similarity) within the orthopoxvirus genus, allowed to achieve cross protection against variola virus by immunization with vaccinia virus (VACV) vaccine strains. In animal studies, vaccination with VACV protects mice against virulent mouse-adapted VACV (WR), ectromelia (ECTV) and cowpox (CPXV) viruses, rabbits from rabbitpox virus (RPXV) and monkeys from monkeypox virus (MPXV) [2].

Although VACV is an efficient vaccine, rare but severe side effects are associated with the vaccine. Along with the success of the eradication campaign, vaccination that was no longer required ceased. As smallpox remains a potential hazard, highly attenuated vaccine strains – e.g. MVA and LC16m8 are being extensively evaluated for potency. In addition, the approach of using protective VACV antigens is pursued by several labs, aiming at generating an efficacious and safe subunit vaccine. In this regard, A33 antigen is one of the most studied and promising candidates [3-13].

A33 is a type-II integral membrane glycoprotein, forming a disulfide-bonded homodimer modified with N- and O- linked glycosylations and acylation and found on the surface of extracellular virions (EV) and infected cells [14-17]. This protein was shown to form a complex with the viral proteins A36, A34 and B5, thus stabilizing their presentation on EVs [16,18,19]. A33 is crucial for virus egress and cell-to-cell spread, and deletion of its gene, namely A33R, results in a small plaque phenotype and virus attenuation in-vivo [20-22]. Antibodies against A33 inhibit virus spread in cell culture as assayed by inhibition of comet formation [22,23].

Using A33-based protein or DNA vaccines, several studies demonstrated the contribution of A33 to protection against VACV, ECTV and MXPV [3-13]. Moreover, antibodies to A33 have a major contribution to protection against VACV challenge [23,24].

In this work we used a recombinant Sindbis virus vector, based on the Toto 1101 attenuated strain that efficiently infect and replicate in mice without associated morbidity or mortality. This replicating virus expresses recombinant proteins cloned downstream to a potent 2nd sub-genomic promoter and therefore induces a robust immune response in mice [25]. In this work, we evaluated the efficacy of a Sindbis virus vector expressing the vaccinia virus protein A33 (A33^VACV^) in protection against several orthopoxviruses. Using this system we show that A33 vaccination protected mice against VACV-WR and ECTV but failed to protect against CPXV challenge. By introducing A33^CPXV^ domains and residues into A33^VACV^, we show that protection with A33^VACV^ based vaccination depends on a single protective epitope in A33^VACV^, previously demonstrated *in-vitro* to serve as a target for the neutralizing antibody MAb-1G10 [26,27]. We further map the role of protective residues and elaborate on the mechanism of cross-protection against orthopoxviruses.

## Results

### Applicability of Sindbis virus as a vaccine vector

**Figure 1:**
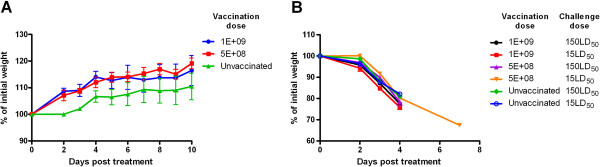
**BALB/c mice tolerate Sindbis pTE vaccination and remain sensitive to subsequent VACV challenge.** BALB/c mice were injected intraperitonealy with 5E + 8 or 1E + 9 pfu of Sindbis pTE or left unvaccinated (as indicated) and monitored daily for any signs of illness. Percent of initial weight profile over 10 days post vaccination is shown (**A**). 14 days post vaccination the mice (either previously vaccinated with 5E + 8 or 1E + 9 pfu of Sindbis virus pTE or unvaccinated) were challenged by intranasal instillation with VACV-WR (15 or 150 i.n. LD_50_) and monitored for any signs of illness. Percent of initial weight profile over 7 days post challenge is shown (**B**) (n = 5 per group). Error bars represent standard errors of the mean (SEM).

In order to evaluate the protective capacity of A33^VACV^, we tested the feasibility of using an expression vector based on a replication competent, recombinant Sindbis virus (pTE3) [25]. First, we assessed the effect of vaccinating mice with this replicating vector and then we tested their susceptibility to subsequent VACV infection. To achieve these goals we vaccinated mice by intraperitoneal (i.p.) injection with two doses (5E + 8 and 1E + 9 pfu) of the parental Sindbis virus (Figure 1A). Vaccination did not cause visible signs of illness with vaccinated mice gaining weight similarly or slightly faster than unvaccinated mice (differences in AUC are not significant p > 0.05) (Figure 1A). No additional visible signs of illness were observed, suggesting low toxicity or reactogenicity of the vector in vaccinated mice. Next, we determined the effect of vaccination with the parental Sindbis virus on the susceptibility of the vaccinated mice to subsequent intranasal (i.n.) lethal challenge with VACV-WR of either 15 or 150 LD_50_. All animals lost weight and succumbed to infection regardless of previous vaccination with Sindbis virus (Figure 1B). These results confirmed the applicability of this virus-based vector for the evaluation of the protective efficacy of A33^VACV^.

**Figure 2:**
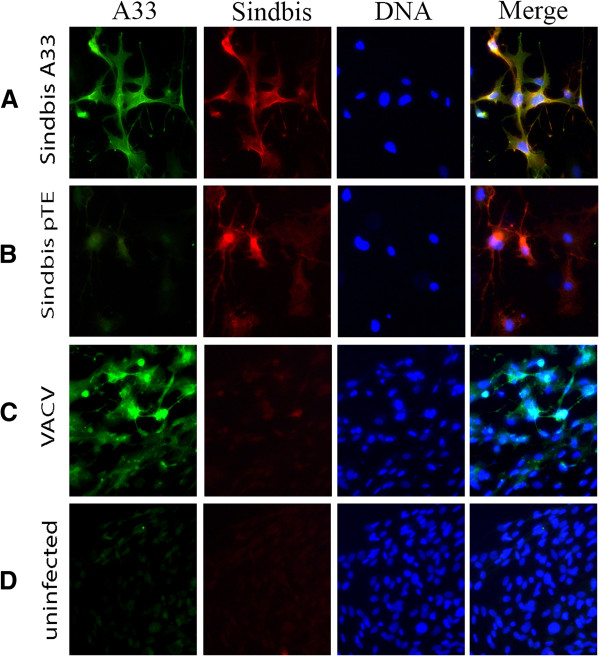
**Expression of A33 in BHK21 cells following infection with Sindbis A33**^**VACV**^**.** BHK21 monolayers seeded on glass cover slides were infected at 0.1 MOI with either Sindbis A33^VACV^ (**A**), Sindbis pTE (**B**), VACV-Lister virus (**C**) or left uninfected (**D**). 24 hours later the cells were fixed, permeabilized and stained with rabbit anti A33 and mouse anti Sindbis virus antibodies, followed by staining with goat anti rabbit antibody conjugated to a 488 nm fluorophore and goat anti mouse antibody conjugated to a 555 nm fluorophore, respectively. Cellular DNA was stained with DAPI.

To confirm the ability of the Sindbis A33^VACV^ vector to express A33 in infected cells, BHK-21 cells were infected with the recombinant virus and A33 expression was determined 24 hours later by immunofluoresence microscopy. Positive staining for A33 was obtained in Sindbis-A33 infected cells (Figure 2A) as well as in the control VACV infected cells (Figure 2C) but not in cells infected with the control parental virus (Sindbis pTE) (Figure 2B) or in uninfected cells (Figure 2D), where only faint non-specific staining was observed. Staining for Sindbis virus in cells infected with Sindbis confirmed the infection efficiency in both samples (Figure 2A and B).

### Sindbis A33 protects against VACV-WR and ECTV challenge

To evaluate the protective efficacy of Sindbis A33^VACV^, BALB/c mice were vaccinated by single i.p. injection with 10^7^pfu of Sindbis A33^VACV^ or Sindbis pTE as a negative control. Fifteen days post vaccination animals were challenged with orthopoxviruses.

**Figure 3:**
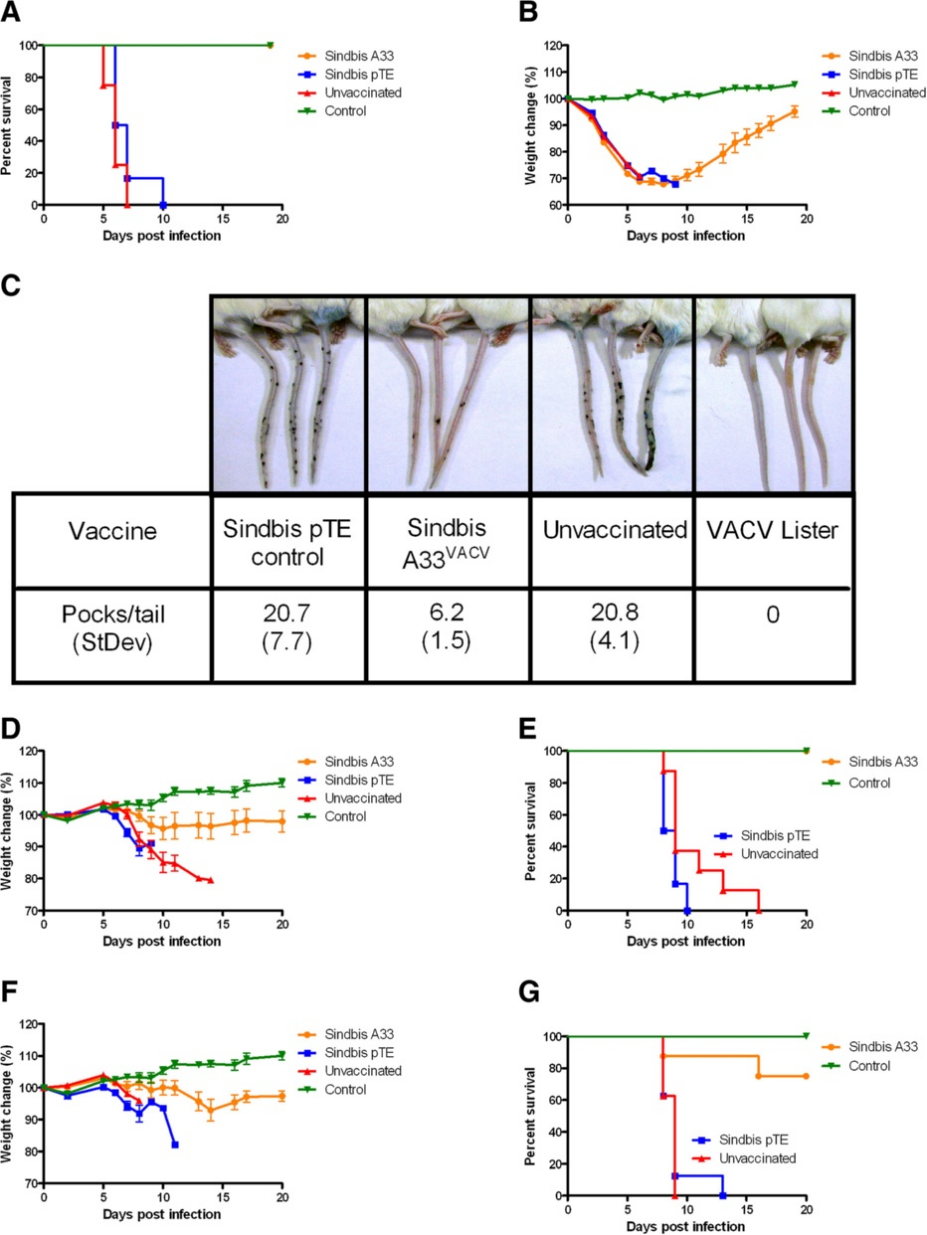
**Protection against orthopoxvirus exposure by Sindbis A33**^**VACV**^**.** BALB/c mice were vaccinated intraperitonealy with 1E + 7 pfu of the indicated viruses: Sindbis A33^VACV^, Sindbis pTE or left unvaccinated. Where indicated (Figure 3C), mice were vaccinated by tail scarification with 1X10^6^ VACV-Lister. Two weeks later, the mice were challenged with VACV^-^WR (4E + 6 pfu i.n. corresponding to 200LD_50_) and monitored for mortality (**A**) and weight loss as a measure of morbidity (**B**). Pocks on the tails were induced in vaccinated or unvaccinated CD-1 mice 14 days post vaccination by intravenous injection with 1E + 04 pfu of VACV-WR (**C**). Pocks on the tails were stained with Trypan blue and counted. Vaccinated mice were challenged with ECTV (10 pfu i.n. corresponding to 10LD_50_) (**D**, **E**) or ECTV by i.f.p. injection (30 pfu corresponding to 3 LD_50_) (**F**, **G**). Mice were weighed daily to monitor morbidity and checked for mortality (n = 6 per group for Figures 3 A & B, n = 5 per group for Figure 5C and n = 7 per group for Figures 3D, E, F & G). Error bars represent standard errors of the mean (SEM).

When VACV-WR, the commonly used mouse adapted strain was used to challenge the mice by intranasal (i.n.) instillation (lethal dose of 4X10^6^pfu corresponding to 200LD_50_), all A33^VACV^ vaccinated mice survived while control Sindbis pTE vaccinated mice and unvaccinated animals succumbed to infection (Figure 3A). Although Sindbis A33^VACV^ vaccination prevented mortality, it did not prevent morbidity (Figure 3B). A33^VACV^ vaccinated mice lost weight similarly to pTE control vaccinated mice. Yet, at day 8 post challenge, A33^VACV^ vaccinated mice began to recover while all control animals died (Figure 3B).

Intravenous infection with VACV-WR leads to a disease visually manifested by pock formation on the tails of the infected mice that can be prevented by vaccination with VACV, as we have shown previously [28]. We used this model to evaluate the protective efficacy of Sindbis A33^VACV^. We vaccinated CD-1 mice with 10^7^pfu Sindbis A33^VACV^ and 14 days later we challenged these animals with 10^4^pfu VACV-WR by i.v. injection. Control pTE and unvaccinated animals exhibited multiple pocks on their tails (Figure 3C). In contrast, Sindbis A33^VACV^vaccination significantly reduced pock formation by 70% (P < 0.001). Intradermal scarification with the conventional smallpox vaccine VACV-Lister completely prevented pock formation. Thus, our data demonstrate that single vaccination with Sindbis A33^VACV^ prevents death and reduces the number of dermal pocks caused by VACV-WR infection.

We then challenged the Sindbis virus based vaccine with ECTV – the causative agent of mousepox which is considered the best small animal model for human smallpox [29,30]. BALB/c mice are highly susceptible to ECTV infection either by i.n. instillation or by intrafootpad (i.f.p) injection. We determined the LD_50_ values for BALB/c as: i.n. LD_50_ = 1pfu and i.f.p LD_50_ = 10pfu ([29] and our unpublished data).

We vaccinated BALB/c mice with Sindbis A33^VACV^ and 14 days later challenged them with 10 i.n-LD_50_ or 3 i.f.p-LD_50_ ECTV (Figure 3D, E and Figure 3F, G, respectively). Following i.n. challenge, control unvaccinated mice or pTE vaccinated animals started to lose weight after 6 days, gradually deteriorated and succumbed to infection within two weeks (mean time to death (MTTD) of 10.5 and 8.7, respectively). In contrast, vaccination with Sindbis A33^VACV^prevented mortality and significantly reduced the extent of weight loss (p < 0.01 comparing the area under the curve of A33 vaccinated vs pTE or unvaccinated mice, Figure 3D).

A33^VACV^ also protected against 3 i.f.pLD_50_ challenge conferring 80% protection from lethality and reducing the extent of weight loss (Figure 3F, G). In contrast, control Sindbis pTE vaccinated mice or unvaccinated mice deteriorated faster and did not survive the challenge.

### CPXV escapes A33^VACV^ induced immunity

CPXV is a rodent orthopoxvirus found in nature in the northern parts of Europe and Asia and occasionally causes emerging zoonosis [2,31,32]. This virus causes a lethal disease in lab mice that can be prevented by smallpox vaccination [28].

**Figure 4:**
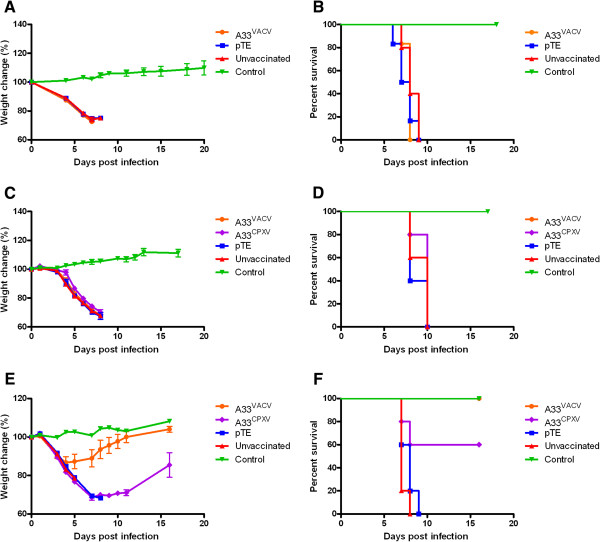
**A33 vaccination does not protect against Cowpox virus.** Mice were vaccinated with 1E + 7 pfu of the indicated viruses: Sindbis A33^VACV^, Sindbis pTE or left unvaccinated. Two weeks later, the mice were challenged with CPXV Brighton-red (3E + 5 pfu corresponding to 3 i.n. LD_50_) and monitored for morbidity (**A**) and mortality (**B**). (n = 5). In a different experiment mice were similarly vaccinated with an additional group vaccinated with Sindbis A33^CPXV^. Two weeks later, the mice were challenged with CPXV Brighton-red (3E + 5 pfu corresponding to 3 i.n. LD_50_) (**C**, **D**) or VACV-WR (4E + 5 corresponding to 20 i.n. LD_50_) (**E**, **F**). Uninfected mice served as a control. Error bars represent standard errors of the mean (SEM).

To measure the potency of A33^VACV^ based vaccine against CPXV infection we vaccinated BALB/c mice with Sindbis A33^VACV^ and 14 days later challenged them with a lethal dose of CPXV (3 i.n. LD_50_). In contrast to the efficient protection against VACV-WR and ECTV, A33^VACV^ vaccinated mice were not protected from a lethal challenge with CPXV (Figure 4A, B). All mice lost weight and died at a similar rate regardless of vaccination. To check whether the basis for the failure of A33^VACV^ to protect against CPXV is sequence variation in A33, we generated a recombinant Sindbis virus encoding the CPXV ortholog of A33 (A33^CPXV^). Similar to vaccination with A33^VACV^, vaccination with A33^CPXV^ neither protected nor prevented morbidity against 3 i.n. LD_50_ challenge of CPXV (Figure 4C, D), excluding the possibility that sequence variation prevents A33^VACV^ from protecting against CPXV challenge. When we tested the ability of A33^CPXV^ to protect against VACV-WR challenge (20 i.n. LD_50_), A33^CPXV^ vaccinated mice were morbid and 40% succumbed to infection whereas all A33^VACV^ survived and were less morbid (Figure 4E, F). All control Sindbis pTE vaccinated and unvaccinated mice succumbed to the infection. Thus, the data indicate that CPXV evades the protective immune response induced by A33^VACV^ or by A33^CPXV^. We further show that unlike A33^VACV^, A33^CPXV^only partially protects against VACV-WR challenge, suggesting that protective epitope(s) in A33^VACV^ that confer protection against VACV and ECTV are modified in A33^CPXV^ making A33^CPXV^ less protective.

### Mapping the protective region in A33

**Figure 5:**
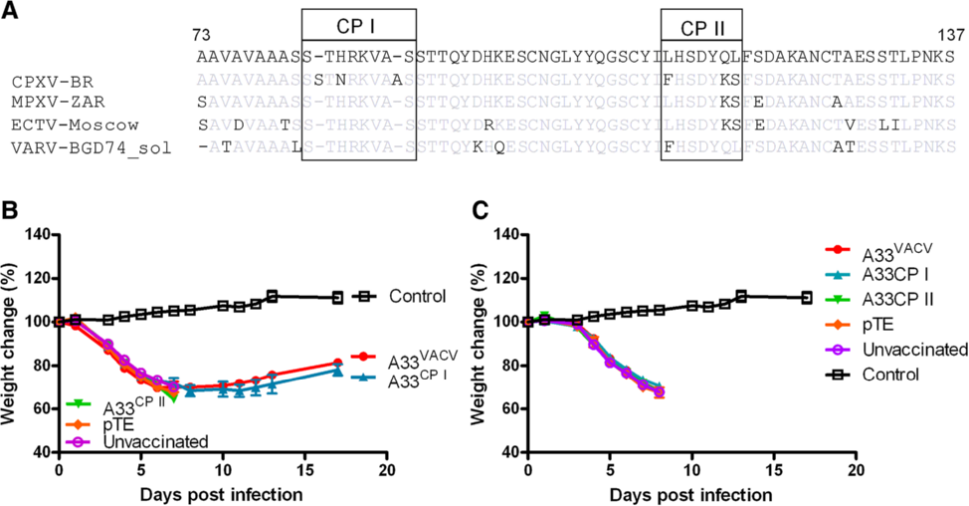
**Mapping the protective epitope to the CP II region in A33.** Alignment of the amino-acid sequence (aa 73–137) of A33^VACV^of: VACV-WR (Vaccinia virus – Western Reserve) with the ortholog A33 of CPXV-BR (CPXV Brighton red), MPXV-Zar (monkeypox Zaire), ECTV-Moscow and VARV-BGD74-sol (Bangladesh 74). CP I and CP II represent two regions with variable changes between the sequences (**A**). BALB/c mice were vaccinated with 1E + 7 pfu Sindbis viruses expressing A33^VACV^, A33^CPXV^ or with A33^VACV^ in which the CP I or CP II regions from A33^CPXV^ were introduced into its genome replacing the original regions (domain swapping). Two weeks later, the mice were challenged with either VACV-WR (4E + 5 pfu corresponding to 20 i.n. LD_50_) (**B**) or with CPXV Brighton-red (3E + 5 pfu corresponding to 3 i.n. LD_50_) (**C**) (n = 5 per group). Error bars represent standard errors of the mean (SEM).

To elucidate the mechanism underlying the inability of A33 to protect against CPXV, we looked for differences in A33 between A33^VACV^ and A33^CPXV^. Additional sequences of monkeypox Zair (MPXV-ZAR), ectromelia virus Moscow (ECTV-Moscow) and Variola major virus starin Bangladesh (VARV-BGD74_sol) were included to elaborate on the role of these sequence variations in pathology of virulent orthopox and protection capacity of molecular vaccines. Comparison of the amino acid (aa) sequences of VACV and CPXV revealed two main regions harboring differences between VACV and CPXV encompassing aa S82-S89 and L112-L118 which we termed CP-I and CP-II, respectively (Figure 5A). The aa changes in CP-I region are unique to CPXV while changes in CP-II appear also in the listed species. The aa sequence of A33 is identical for VACV-WR (the challenge strain) and for VACV-Lister (the vaccine strain). In order to elaborate on the role of these two regions in protection, two chimeric molecules were constructed by replacing the A33^VACV^ CP-I and CP-II regions with the corresponding CPXV sequences. The chimeric A33 genes were introduced to the Sindbis expression system. Mice were vaccinated with the recombinant Sindbis virus encoding A33^VACV^, A33^CPXV^ or the chimeric A33 proteins, and 14 days post vaccination were challenged by intranasal instillation with either VACV-WR (20LD_50_) or CPXV (3LD_50_). Replacement of the A33^VACV^ backbone with A33 CP-I region did not affect the inherent protective capacity of A33^VACV^ and the mice recovered similarly to A33^VACV^ vaccination (Figure 5B). However, replacement of the A33^VACV^ backbone with A33 CP-II region resulted in loss of protection against VACV-WR challenge (Figure 5B). None of the tested constructs provided protection against CPXV challenge (Figure 5C). The loss of protection that resulted from replacement of the CP-II region of VACV with the corresponding CPXV region, suggests that CP-II harbors protective epitope(s). This epitope is not protective in the corresponding CP-II region of CPXV.

### Identification of the critical residues for protection in A33

**Figure 6:**
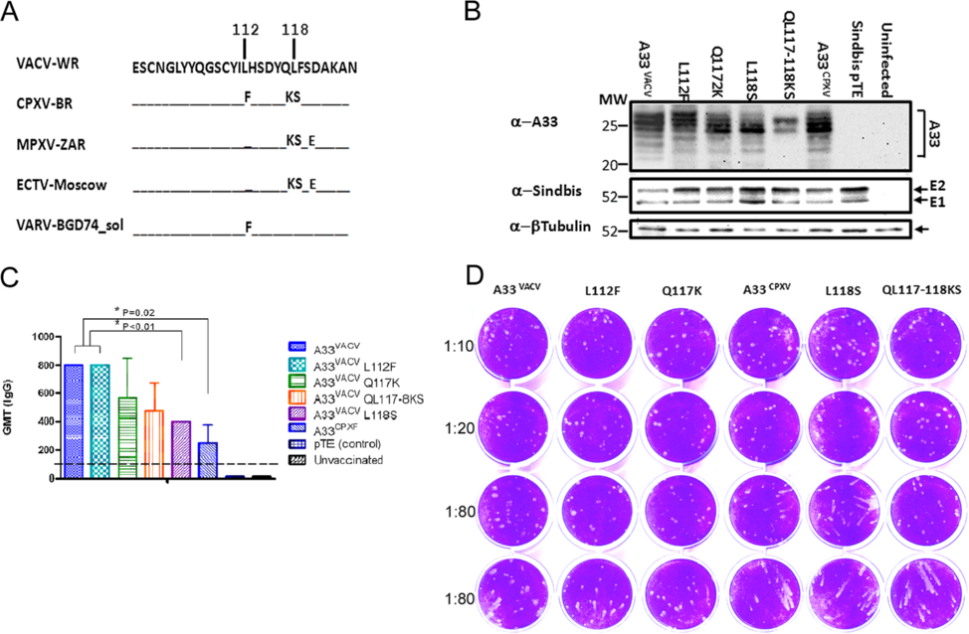
**Expression and induction of antibodies with comet inhibition activity by Sindbis A33 harboring point mutations within the CP II region.** Multiple sequence alignment of A33 CP II region showing amino acid changes between several Orthopoxviruses-VACV-WR (Vaccinia virus – Western Reserve), CPXV-BR (CPXV Brighton red), MPXV-Zar (monkeypox Zaire), ECTV-Moscow and VARV-BGD74-sol (Bangladesh 74) (**A**). Expression analysis of the various A33 genes in lysates of Sindbis A33 infected BHK21 cells separated by SDS-PAGE and immunoblotted as indicated. MW – Molecular weight (Kda). Arrowheads indicate bands of the different proteins (**B**). Geometric mean titer (GMT) (ELISA) in sera of vaccinated mice 21 days post vaccination. Bars represent GeoStDev (**C**). Comet inhibition activity in sera of Sindbis A33 vaccinated mice (**D**, same sera as in **C**). Serum dilutions are indicated to the left and the type of Sindbis A33 used for vaccination is indicated at the top.

Next, we wanted to identify which of the residues in CP-II contribute to protection. The CP-II region in A33^CPXV^ harbors the following amino acid substitutions compared to A33^VACV^: L112F, Q117K and L118S (Figure 6A). The L112F substitution is shared also by variola virus (VARV-BGD74_sol) while the Q117K and L118S substitutions are found in monkeypox (MPXV-ZAR) and ectromelia (ECTV-Moscow) but not in VARV. Both MPXV and ECTV share also the S120E substitution. To check which of these substitutions abrogates protection, we generated modified A33^VACV^ genes harboring the L112F, Q117K and L118S substitutions on the backbone of A33^VACV^ . In addition, we also generated a double mutant A33 gene (Q117K-L118S). These modified A33^VACV^ genes were cloned into the Sindbis expression system. Recombinant Sindbis viruses were produced and expression of the modified A33 genes was confirmed by infection of BHK 21 cells followed by Western blot analysis of the cell lysates (Figure 6B). A33 is post-translationally modified in cells (N’ and O’ glycosylations and acylation) resulting in a well-known multiple band pattern in SDS-PAGE [14,16,19]. All Sindbis viruses expressed the modified A33^VACV^ genes, yet certain differences in the multiple band pattern could be observed, mainly in the double mutant A33 (Q117K-L118S). Expression of the E1 and E2 sindbis virus proteins and β-Tubulin confirm the specificity of A33 detection and serve as infection and loading controls.

Next, we evaluated the effect of the aa substitutions on the capacity of A33 to induce immune response and protection. To analyze the effect of the aa substitutions on ability of A33 to mount cytotoxic CD8+ T cell lymphocyte (CTL) dependent cytotoxicity, we performed *in-silico* analysis of A33 potential CTL epitopes utilizing 3 independent experimentally based algorithms: NetMHC, SYFPEITHI and BIMAS [33-35]. Since evaluation of the protective ability of A33 is performed in BALB/c mice, the analysis was set to predict epitopes that can bind the BALB/c MHC class I molecule (H-2Kd and H-2Dd alleles). All three algorithms identified a single H-2kd prevalent 9-mer potential CTL epitope in the entire A33^VACV^gene spanning aa Y104 to L112, in which the essential anchoring residue L112 is located at the CP-II region. In addition, all three algorithms predicted that the 9–mer peptide harboring the L112F substitution, representing the CPXV orthologous peptide, would lose the ability to bind the MHC molecule, a prerequisite to elicit CD8+ T-cell cytotoxicity.

To check whether the 9-mer epitope in CPII harboring L112 is a functional CTL epitope, and whether the L112F substitution abrogates it, BALB/c mice were vaccinated with sindbis A33^VACV^ and 14 days later were tested for their ability to mount *in-vivo* CTL activity (Table 1). Vaccination with VACV-Lister, the licensed smallpox vaccine and the H-2Kd immune dominant T-cell epitope of VACV A52 [36] were used as positive controls. Naïve mice and the unrelated influenza virus H-2Kd CTL epitope (Influenza A/H3N2/Texas/1/77 NP 147–158) [37] were used to calculate specific lysis. In support of previous reports [36], VACV-Lister vaccinated mice mounted about 40% specific lysis of A52 labeled cells unlike naïve, unvaccinated mice. The predicted 9-mer H-2Kd CTL epitope spanning aa Y104 to L112 of A33^VACV^ did not mount significant specific lysis neither following sindbis A33^VACV^ vaccination nor following VACV-Lister vaccination. Interestingly, peptide harboring L112F substitution, predicted to prevent CTL activity, mounted a weak, yet significant response. In summary, our results do not support the presence of a functional H-2Kd CTL epitope spanning aaY104 to L112. Therefore, the data does not support the hypothesis that immune evasion of A33 by CPXV is due to the loss of a CTL epitope through the L112F substitution.

**Table 1:** ***In vivo *CTL activity to A33 peptides in vaccinated mice**

**Vaccination**	**Specific peptide**	**% Specific lysis (StDev)**
Sindbis A33^VACV^	A33^VACV^ (104–112)	0 (1.3)
VACV Lister		2.5 (2.9)
Sindbis A33^VACV^	A33^CPXV^ (104–112)	6.1 (2.8)
VACV Lister	A52^VACV^ (75–83)	40.4 (6.3)

*In vivo* CTL activity to the indicated peptides (middle column) was measured 14 days following vaccination of BALB/c mice with the indicated viruses (left column). Specific lysis (% and StDev) is depicted in the right column.

We then tested the level of serum IgG antibodies against A33 following vaccination with the various A33 derivatives. All A33 derivatives induced specific antibodies as determined by ELISA using A33^VACV^ as the capture antigen (Figure 6C). Yet, lower antibody titers were detected following vaccination with A33^CPXV^ or the L118S substitution (P = 0.02 and P < 0.01 compared to A33^VACV^/A33^VACV L112F^, respectively). As protective antibodies against A33 were previously demonstrated to inhibit EV spread in culture, we analyzed the capacity of the different sera to inhibit EV spread assayed by comet formation inhibition. A33^VACV^ as well as the L112F or Q117K mutants inhibited comet formation. In contrast, sera from A33^CPXV^ or L118S vaccinated mice poorly inhibited comet formation (Figure 6D). Comet inhibition by serum from the double substitution Q117K - L118S vaccinated mice inhibited EV spread only partially.

**Figure 7:**
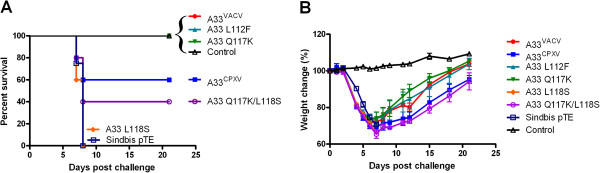
**Mapping the protective epitope in A33.** Mortality (**A**) and morbidity (**B**) in mice vaccinated with various A33 constructs. BALB/c mice were vaccinated with 1E + 7 pfu of Sindbis viruses expressing the various A33 proteins or with a control Sindbis pTE as indicated. 14 days later the mice were challenged by i.n. instillation with VACV-WR (4E + 5 pfu corresponding to 20 LD_50_). (n = 5 per group). Error bars represent standard errors of the mean (SEM).

To elucidate the protective capacity of these variations, vaccinated mice were challenged by intranasal instillation with VACV-WR (20 LD_50_, Figure 7). Whereas L112F and Q117K substitutions protected mice similarly to A33^VACV^ (100% survival), L118S substitution resulted in complete loss of protection (Figure 7A). The Q117K - L118S double substitution resulted in partial protection (40% survival), and the recovery rate of the surviving animals in this group was slower in comparison to A33^VACV^ vaccinated mice (p < 0.004) (Figure 7B). Thus, our data indicates that of the several amino acid differences between the A33^VACV^ and A33^CPXV^, L118 as part of a major protective epitope, significantly contributes to the protection afforded by active vaccination with A33, most likely through induction of protective humoral immunity.

## Discussion

In the present study we evaluated the applicability of a Sindbis-virus based vector for the evaluation of the protective efficacy of A33^VACV^. Previous reports demonstrated the protective capacity of A33 against different orthopoxviruses such as VACV-WR, ECTV and MPXV [3-8,13]. We show that a single-dose vaccination of Sindbis A33^VACV^ efficiently protected mice against virulent VACV-WR and ECTV challenge, but failed to protect against CPXV challenge. Furthermore, homologous vaccination with A33^CPXV^ failed to protect against CPXV and only partially protected against VACV-WR. We identified a single protective region in A33^VACV^ located between residues 104–120 that harbors a putative H-2Kd CD8+ T cell epitope and a B cell epitope. This B cell epitope was shown to be a target for the neutralizing antibody MAb-1G10 that blocks spreading of extracellular virions [26,27]. Both epitopes are affected in A33^CPXV^ by 3 amino acids (aa) substitutions L112F, Q117K and L118S and shown to be non-functional. Vaccination with a A33^VACV^, modified to contain each of these substitutions, revealed the critical role of L118 in protection against VACV-WR and in blocking of EV spread by antibodies. These results support previous studies which mapped the critical role of L118 in binding the monoclonal neutralizing antibody MAb-1G10 and the consequence of each of these substitutions on binding of this antibody [26,27]. Since A33 is considered an important contributor to future smallpox subunit vaccines which might also be used against other orthopoxvirus infections such as MPXV, and antibodies to A33 are developed for treatment of orthopoxvirus infections, unraveling the basis for this protective ability at the single aa level is of major importance.

*In-silico* analysis of A33 identified a potential CTL epitope in which L112 is a pivotal anchoring residue. L112F substitution representing the CPXV orthologous peptide, abolished the predicted binding to the H-2Kd molecule thus impairing the ability of A33 to elicit a cellular immune response. The very same substitution is also found in the VARV homologue A33^VARV^ (Figure 6A) making this specific residue of interest. In contrast to the *in-silico* predictions, A33 vaccination did not induce CTL activity to the 9 mer Y104 to L112 predicted epitope of A33^VACV^ (Table 1). Surprisingly the L112F substituted epitope (found in A33^CPXV^) induced a low level of CTL activity, yet it did not correlate with *in-vivo* protection. Since L112F substitution retained the protective ability of A33 we conclude that at least in the BALB/c mouse model, this epitope does not induce T-cell dependent cytotoxicity. As L112F substitution is also found in A33^VARV^, we tested the possibility that this substitution may render VARV resistant to A33-based vaccination. In humans, two overlapping predicted A33^VACV^ CTL epitopes were mapped at this region spanning aa 102–111 (based on the A0201 allele of the HLA-A2 supertype) [38] and 109–118 (NetCTL prediction, CBS). Despite strong *in-vitro* binding of the former epitope (aa 102–111) to A0201 allele, CTL activity (IFNγ release) was not detected [39]. Additionally, the L112F variation found in A33^VARV^ is not predicted to affect the anchoring to the A0201 molecule. Thus, our experimental data and previous studies do not support the presence of a functional CTL epitope within the CP-II region, suggesting that F112 substitution found in A33^VARV^ most probably will not abrogate protective immunity through vaccination with A33^VACV^.

We evaluated several single aa substitutions in the CP-II region and demonstrated that L118 is the only residue which is crucial for protection against VACV-WR. These results are in complete agreement with the mapping of the binding epitope of the protective/neutralizing monoclonal antibody MAb-1G10 [26]. In that study, the L118S change in MPXV was shown to be detrimental for the MAb-1G10 binding and the Q117K substitution partially restored this binding [26]. In our hands, sera from mice vaccinated with an A33 harboring the L118S substitution exhibited a lower antibody titer reflected by reduced comet inhibition potency. It was previously demonstrated that comet inhibition activity correlates with protection against lethal VACV-WR challenge [40]. It appears that the inability of A33 to protect BALB/c mice against CPXV challenge, most likely results from the inability of the A33^VACV^-induced protective antibodies to recognize A33^CPXV^. It is possible that the L118S substitution, replacing a non-polar hydrophobic with a polar-hydrophilic residue, interferes with antibody binding to the epitope containing L118.

We showed that vaccination with A33^CPXV^ did not protect against CPXV challenge and only partially protected against VACV-WR challenge (Figure 4). These results support the study mentioned above, using the MPXV ortholog of A33 (A33^MPXV^) which also partially protected against VACV-WR challenge [26]. Unlike CPXV, both MPXV and ECTV encode the E120 residue that is believed to compensate for the L118S substitution found in A33^MPXV^ and A33^ECTV^ (Figure 6A). In this work we further show the critical role of L118 in protection against lethal challenge with VACV-WR by active vaccination. Having the same mouse model for VACV-WR, CPXV and ECTV, allowed us to evaluate the differential cross-protection capacity of A33, and allowed to demonstrate the critical role of L118 and the compensatory role of Q117 and E120.

The results also agree with previous work by He et al. [27], substantiating the essential role of L118 in binding of protective antibodies to A33. In that work, an important role in antibody binding to the epitope is attributed to the D115 residue, yet D115 is conserved between VACV and CPXV excluding its contribution to the mechanism utilized by CPXV to evade A33 immunity. Our work shows that active vaccination with A33 induces immunity to a single protective epitope and suggests that the mechanism of protection involves induction of MAb-1G10 like neutralizing antibodies. This epitope overlaps an immunogenic region in A33, previously mapped in primates to mount antibody response [5,24,41], further emphasizing the importance of this region for future vaccines.

We show that the L118S substitution abrogated protection, which was partially restored, by the double mutant Q117K-L118S. This correlates with partial restoration of the binding of MAb-1G10 antibodies to A33 by the Q117K substitution [26]. This suggests that beside the major L118 residue, other residues, within the protective epitope, contribute to protection. Indeed, A33^VACV^ protects BALB/c against ECTV challenge. A33^ECTV^ bares the same Q117K-L118S substitutions, and similarly to MPXV has an additional S120E substitution, not found in CPXV. This substitution was shown to improve binding of the MAb-1G10 neutralizing antibody [26], pointing toward E120 as a compensatory residue. It is possible that boosting immunization with A33 enhances an immune response through additional weak epitopes that support protection in very low challenge doses of CPXV [42].

Having demonstrated that replacement of CP-II eliminates the protection afforded by A33 and that CP-I replacement was as protective as A33^VACV^ it was reasonable to suggest that a major protective epitope is located within CP-II, as demonstrated in the manuscript. Whether CP-I replacement synergize with CP-II cannot be simply tested because CP-II of CPXV was already associated with 100% lethality. Nevertheless, based on the partial protection against VACV-WR afforded by vaccination with A33^CPXV^ we cannot exclude the possibility that the CP-I region harbors a compensatory function to the CP-II region. This possibility was not tested.

CPXV utilizes several mechanisms of immune evasion including inhibition of TAP dependent antigen processing, inhibition of MHC class I peptide loading, inhibition of cell surface presentation of MHC class I molecules and expression of large set of immune modulating genes [43]. Whether modifying a few residues within a major protective epitope prevents the binding of neutralizing antibodies to A33 thus allowing CPXV to evade A33 immunity, remains to be demonstrated.

The immune protection conferred by VACV and its antigens, against VARV and other orthopoxviruses is based on cross-protection. This fact, and the development of subunit vaccines and monoclonal antibody-based therapeutic products, strongly suggest that sequence variation between the species should be carefully assessed. Mapping the basis for the protective ability of A33 at the single amino acid level, as conducted in this study, further highlights this aspect of cross-reactivity and sequence variation that affect protection.

## Materials and methods

### Viruses, cells and antibodies

Vero (ATCC-CCL-8), BHK 21 (ATCC-CCL-10) RK-13 (ATCC-CCL-37) and BS-C-1 (ATCC-CCL-26) cells were maintained as recommended by ATCC. Vaccinia Lister (Israel Ministry of Health), VACV-WR (ATCC VR-119), CPXV strain Brighton-red (ATCC VR-302) and IHD-J were propagated and tittered as described previously [28]. Sindbis virus (pTE32J) was prepared as described [25]. Briefly, capped pTE32J RNA was generated by *in-vitro* transcription using SP6 RNA polymerase and transfected to BHK-21 cells using Lipofectin (invitrogen) according to the manufacturer protocol. Viruses were collected from the culture medium 48 hours post transfection, reinfected to BHK-21 cells and the viruses were collected from the culture medium, concentrated by the slow addition of Polyethylene-glycol (PEG) 8,000 and NaCl (Sigma) to reach a 40% PEG 2 N NaCl concentration. Following 60 minutes incubation at 4°C, viruses were pelleted at 11,000 g for 60 minutes at 4°C, and the pellet was resuspended in PBS. The concentrated viruses were titrated on BHK cells and maintained in aliquots at −80°C. A33 antisera was generated as described [44]. Mouse anti β Tubulin antibody was purchased from Sigma. Mouse and rabbit anti Sindbis antisera were generated by repeated vaccination of mice and rabbits with Sindbis virus. Rabbit anti A33 was obtained by repeated vaccination with bacterially expressed, affinity purified, His-tagged A33.

### DNA sequences and CTL predictions

The following sequences were used for DNA sequence alignments: VACV-WR (Vaccinia virus WR, accession# NC006998), CPXV-BR (Cowpox virus Brighton-red, NC003663), VACV-Lister (AY678276), ECTV-Moscow (Ectromelia virus Moscow, NC004105), MPXV-ZAR (Monkeypox virus Zair, NC003310) and VARV-BGD74_sol (Variola virus Bangladesh 1974, DQ441422). Prediction of CTL epitopes were performed using NetMHC (http://www.cbs.dtu.dk/services/NetMHC), SYFPEITHI (http://www.syfpeithi.de/bin/MHCServer.dll/EpitopePrediction.htm), and BIMAS (http://www-bimas.cit.nih.gov/molbio/hla_bind).

### Cloning and expression of A33

A33R gene from VACV Lister or CPXV Brighton-red DNA was amplified and cloned to the pH3 shuttle vector and transferred to the pTE3 vector [25]. Point mutations and domain swapping were generated by PCR, and validated by DNA sequencing. Expression of A33 in BHK-21 infected cells was determined by immunofluorescence microscopy and western blotting as previously described [45] using rabbit anti-A33 antibody, mouse anti-Sindbis antibody and anti β-Tubulin (Sigma) antibody. HRP, Alexa-fluor 488 and Alexa-fluor 555 conjugated anti-rabbit and anti-mouse conjugated antibodies (Molecular probes) were used according to the manufacturer’s protocols. DAPI (Sigma) was used according to the manufacturer’s protocols to stain nuclear DNA. Immunofluorescent microscopy was performed using an axiovert Zeiss microscope. Image acquisition was performed, maintaining the same settings for all samples, using a DS-Ri1 CCD (Nikon). Bacterially expressed, His-tagged A33, was used for rabbits immunization. To achieve that goal, A33^VACV^ from VACV Lister was cloned into pRSET plasmid (invitrogen) and expressed in BL21::pLysS bacteria. A33 was purified from the cell lysates on a Nickel-agarose (NiNTA) column (Qiagen) according to the manufacturer’s protocol, analyzed on SDS-PAGE and formulated for vaccination with complete or incomplete Freund adjuvant (Sigma) according to the manufacturer protocols.

### Enzyme linked immunosorbent assay (ELISA)

Antibody titer to Vaccinia virus was determined by ELISA as described [28]. Briefly, microtiter plates were coated with 50 ng purified bacterially expressed 6xHis tagged A33^VACV^. The protein was purified from whole 8 M Urea denatured bacterial lysates on a NiNTA resin (Qiagen) and dialyzed against PBS. Following adsorption, plates were blocked, incubated with two-fold serial serum dilutions and then subsequently with alkaline phosphatase conjugated goat anti mouse IgG (1:1000, Sigma-Aldrich). GMT and GeoSTDEV were calculated as described [28].

### Comet inhibition assay

Comet inhibition assay in mouse sera was determined as described previously [23]. Briefly, fresh preparation of IHD-J, diluted in Earle’s modified Eagle medium, supplemented with 2% heat-inactivated fetal bovine serum to give approximately 50 plaques was used to infect monolayers of BS-C-1 cells. After 2 h at 37°C, the inoculum was removed and the cells were washed. A liquid overlay, consisting of Earle’s modified Eagle medium, supplemented with 2% heat-inactivated fetal bovine serum and serial dilutions of sera obtained from the A33 vaccinated mice as indicated. After 48 h, the medium was aspirated and the cells were stained – fixed with 0.1% (W/V) crystal-violet (Merk) in 20% ethanol. The stain was aspirated and the wells were washed with tap-water and dried. Comet inhibition capacity is determined by the minimal serum dilution exhibiting such activity.

### Mice vaccination and challenge

BALB/c and CD-1 female mice (5–7 weeks old, Charles Rivers, UK) were vaccinated by intraperitoneal injection (i.p) with 1×10^7^pfu of the various Sindbis viruses diluted in phosphate buffered saline (PBS). Intranasal challenge with VACV-WR or CPXV was performed as previously described [28]. To induce pocks on the tails, CD-1 mice were similarly vaccinated with the various Sindbis viruses or with 1×10^6^pfu of VACV-Lister by tail scarification and 14 days later were intravenously injected with 1×10^4^pfu of VACV-WR. 8 days later the tails were stained with 0.1% Trypan blue and pocks were counted. The method of tail scarification as well as the calculation of the lethal dose of 50% for BALB/c mice (LD_50_) were performed as described [28]. The end-points were weight loss of 40% of the initial weight and/or the inability to respond to the righting reflex. Animals that reached these predetermined end-points were humanely sacrificed. LD_50_ values by i.n. instillation in BALB/c mice were determined as: 2×10^4^pfu for VACV-WR, 1×10^5^pfu for CPXV and 1 pfu for ECTV. LD_50_ of ECTV by i.f.p. injection was 10 pfu. Injection i.v. of 1×10^4^pfu VACV-WR to CD-1 mice was not lethal. Animal experiments were repeated at least twice and conducted in compliance with the regulations for animal experiments of the IACUC at the Israel Institute for Biological Research.

### In-vivo cytotoxicity assay

MHC class I–dependent cytotoxicity assay was modified from [46]. In brief, syngeneic splenocytes were harvested from naïve BALB/c mice and labeled with either DiI or DiD (invitrogen) according to the manufacturer manual. DiI labeled cells were pulsed with a vaccinia CD8+ T-cell epitope with the amino acids sequence KYGRLFNEI (corresponding to -A52^VACV^_75-83_) [47] or with the putative K^d^ T-cell epitope from A33^CPXV^_104-112_ (YYQGSCYIF) or with the putative K^d^ T-cell epitope from A33^VACV^_104-112_ (YYQGSCYIL) at a final concentration of 1 μg/ml. DiD labeled cells were pulsed with a T-cell epitope of influenza-A/H3N2/Texas/1/77 NP_147-158_ (TYQRTRALVRTG) [37] as control at a final concentration of 1 μg/ml. A52^VACV^_5-83_ and influenza-A/H3N2/Texas/1/77 NP_147-158_ are restricted to K^d^. DiI and DiD labeled cells were mixed to 1:1 ratio and 2×10^7^ cells were injected into the tail vain of naïve or vaccinated BALB/c mice. 4 hours after injection recipient animals were sacrificed and their spleens were removed. The amount of DiI and DiD labeled cells from the spleens was determined by flow cytometry (FACScalibur, BD) and analyzed with the Flowjo software (Tree Star). Specific lysis was calculated with the following formula:

$$\%\;\mathrm{specific}\;\mathrm{lysis}=\left[1-\frac{\mathrm{ratio}\;\mathrm{unvaccinated}}{\mathrm{ratio}\;\mathrm{vaccinated}}\right]\times 100$$

$$\mathrm{Where}\;\mathrm{ratio}=\frac{\mathrm{\%DiD}\;\mathrm{labelled}\;\mathrm{cells}}{\mathrm{\%Dil}\;\mathrm{labelled}\;\mathrm{cells}}$$

### Statistical analysis

The area under the curve (AUC) to compare morbidity based on weight loss was calculated for each mouse and mean AUC’s of various groups were compared using two-sample Student *t*-test (GraphPad Prism, Irvine, CA). Significance between antibody titers was calculated using two-tailed, unpaired Student *t*-test (GraphPad Prism, Irvine, CA).

## Competing interests

The authors declare that they have no competing interests.

## Authors’ contributions

NP and SL and UO participated in the design of the study, carried out the experiments and drafted the manuscript. AZ performed the predictions of CTL epitopes and helped to draft the manuscript. SM, TI and NE participated in the design of the study and helped to draft the manuscript. DBN, RL, BL, OI, DS and BP helped to carry out the experiments. All authors read and approved the manuscript.
